# Closed-Loop Implantable Therapeutic Neuromodulation Systems Based on Neurochemical Monitoring

**DOI:** 10.3389/fnins.2019.00808

**Published:** 2019-08-20

**Authors:** Khalid B. Mirza, Caroline T. Golden, Konstantin Nikolic, Christofer Toumazou

**Affiliations:** Department of Electrical and Electronic Engineering, Centre for Bio-Inspired Technology, Institute of Biomedical Engineering, Imperial College London, London, United Kingdom

**Keywords:** neurochemical monitoring, closed loop neuromodulation, deep brain stimulation (DBS), vagus nerve stimulation (VNS), FSCV, chemometrics

## Abstract

*Closed-loop* or intelligent neuromodulation allows adjustable, personalized neuromodulation which usually incorporates the recording of a biomarker, followed by implementation of an algorithm which decides the timing (*when?*) and strength (*how much?*) of stimulation. Closed-loop neuromodulation has been shown to have greater benefits compared to *open-loop* neuromodulation, particularly for therapeutic applications such as pharmacoresistant epilepsy, movement disorders and potentially for psychological disorders such as depression or drug addiction. However, an important aspect of the technique is selection of an appropriate, preferably neural biomarker. Neurochemical sensing can provide high resolution biomarker monitoring for various neurological disorders as well as offer deeper insight into neurological mechanisms. The chemicals of interest being measured, could be ions such as potassium (K^+^), sodium (Na^+^), calcium (Ca^2+^), chloride (Cl^−^), hydrogen (H^+^) or neurotransmitters such as dopamine, serotonin and glutamate. This review focusses on the different building blocks necessary for a *neurochemical, closed-loop* neuromodulation system including biomarkers, sensors and data processing algorithms. Furthermore, it also highlights the merits and drawbacks of using this biomarker modality.

## 1. Introduction

The idea of treating intractable diseases with little or no known pharmacological interventions through the nervous system has led to a new area of therapeutic treatment, known as *electroceuticals* or *bio-electronic medicine* (Kristoffer et al., 2013). The therapeutic effects of *electroceutical* techniques are observed by modulating signals on the nervous system through external agents such as electrical stimulation. This process is known as neuromodulation. Current applications of electroceuticals target diseases such as Parkinson's disease (Tass, 2003; Benabid et al., 2009; Ebert et al., 2014), epilepsy (Amar et al., 2008), depression (Landau et al., 2015), diabetes (Shikora et al., 2013), inflammation (Borovikova et al., 2000; Tracey, 2002; Li et al., 2016), auto-immune diseases such as Crohn's disease (Bonaz et al., 2016), regulation of blood pressure (Hosokawa and Sunagawa, 2016) and obesity (Payne et al., 2018).

Closed-loop neuromodulation has been shown to be clinically more effective than open-loop neuromodulation (Sun and Morrell, 2014), under certain conditions. To implement a closed-loop neuromodulation paradigm, important aspects to consider are identifying the relevant neural *biomarker*, identifying the optimal *location(s)* for monitoring the biomarker and electrical stimulation, respectively, implementing the *sensing methodology* and instrumentation for the biomarker, followed by signal processing to differentiate the biomarker responses from background interferences, decision and dose tuning algorithms to determine *when* and *how much* to stimulate.

Traditional biomarkers for closed-loop neuromodulation include electrical neural signals such as action potentials (AP) or local field potentials (LFP), with many devices providing high channel count neural recording and processing (Zhou et al., 2019). There have also been recent efforts to incorporate high-resolution stimulation through optogenetic methods (Mickle et al., 2019). Non-neural biomarkers such as electrocardiography (ECG), electromyography (EMG) signals have also been used, particularly in epilepsy and movement disorders (Sun and Morrell, 2014), either as a direct or as an adjunctive biomarker.

For example, in Parkinson's disease, excessively synchronized neural activity is a crucial sign of Parkinson's. A technique, Coordinated Reset Stimulation (CRS) which seeks to desynchronize this abnormal synchronization by computationally modeling stimulation, is gaining traction. The unique advantage is that, the stimulus could be invasive electrical (Tass, 2003) or even non-invasive sensory stimulation such as somatosensory or vibrotactile stimulation. This review is primarily focussed on neurochemical biomarkers for closed-loop systems.

Neurochemical recording is an emerging form of neural recording, where ionic species or neurotransmitters, present inside neurological systems are monitored. Neurochemical monitoring has multiple advantages over traditional electrical neural recording including higher specificity in comparison to traditional electrical recording of neural activity, lesser interference from other signals such as EMG or heart rate (Cork et al., 2018) and possibility to detect inhibitory and excitatory neural activity by monitoring the concentration of specific neurotransmitters (Wightman et al., 1988).

In recent years, there has been significant traction in the pursuit of neurochemical closed-loop feedback in deep brain stimulation (DBS). A recent National Institute of Health (NIH) grant was aimed at exploring neurochemical recording for DBS applications (NIH, 2014). Another interdisciplinary seed grant was recently awarded by Stanford Bio-X, which is aimed at developing neurochemical closed-loop DBS system for treating psychiatric disorders (Stanford-Bio-X, 2018).

DBS is an invasive electrical stimulation therapy used to treat neurological disorders such as Parkinson's Disease (Krack et al., 2003; Bittar et al., 2005; Beuter et al., 2014), essential tremor (Koller et al., 1999; Rehncrona et al., 2003; Flora et al., 2010), chronic pain (Marchand et al., 2003; Owen et al., 2006; Boccard et al., 2015), and dystonia (Vidailhet et al., 2005, 2013). Although the therapeutic effects of DBS for symptomatic relief in Parkinson's is well appreciated, the inherent mechanisms are still not well understood.

The standard protocol in DBS is to follow a trial and error technique, whereby a given set of stimulation parameters are tested on-the-fly by the neurosurgeon during surgery (Volkmann et al., 2002). For instance, in the case of a patient with essential tremor, the feedback signal is to observe the amplitude of the tremor while the stimulation is on (Volkmann et al., 2002). Although numerous simulations have been performed in modeling the effect of stimulation on the surrounding neural tissue (Yousif et al., 2010, 2012; Golden et al., 2013), one cannot be sure of the nature of excitation or inhibition that is being introduced in the local neural network for a given therapeutic outcome *in vivo*. There are two potential feedback loops within DBS; electrical activity of the neural network, and neurochemical activity. In the former category, there are studies that are advancing the technology to a closed-loop system (Priori et al., 2013; Rosa et al., 2015), whereby the electrical activity of the surrounding population of neurons is used as a feedback signal. However, as this method relies on detecting electrical signals from surrounding neurons, it is highly susceptible to significant stimulation artifacts from the proximal stimulation electrode.

In comparison, a neurochemical feedback system measuring ions and neurotransmitters, has potential to gain a more nuanced picture of the effect of DBS, on the surrounding neural tissue. Indeed, efforts are being made in this endeavor, primarily in the development of the WINCS system (Chang et al., 2013; Grahn et al., 2014), a single channel, wireless, neurochemical feedback system for DBS. This technology has been advanced to a multichannel feedback loop in WINCS Harmoni (Lee et al., 2017), which has so far proven effective in rodents and swine.

Hence, this review is focused on describing the building blocks for a neurochemical closed-loop system. It describes briefly, the different neurochemical biomarkers that could be used in different neurological diseases and the sensing methodologies that have been used for these neurochemicals. It primarily focuses on processing algorithms for decision making and dose-tuning.

In this paper, section 2 describes various neurochemical biomarkers and respective optimal recording/stimulation loci for different neurological disorders. Section 3 describes different sensing methodologies that can be used to monitor various neurochemical biomarkers. The different steps required to implement an intelligent, implantable neuromodulation system are described in section 4. Discussion and Conclusions related to the above mentioned topics are presented in sections 5 and 6, respectively.

## 2. Neurochemical Biomarkers

**Figure 1:**
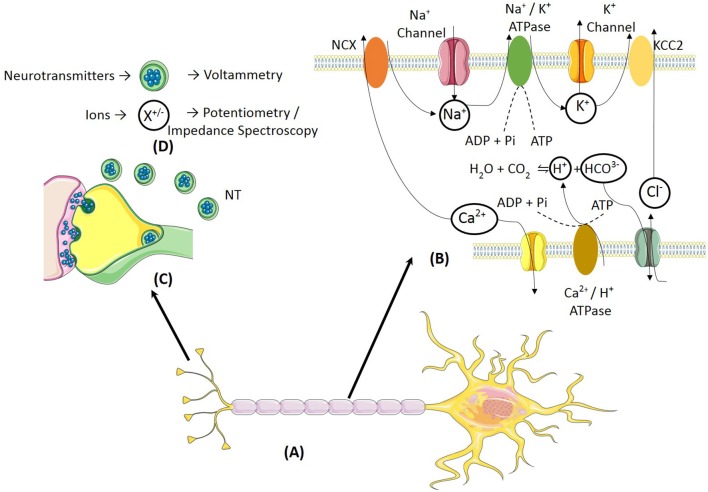
**(A)** A typical neuron shows ionic and neurotransmitter transients induced due to neural activity. **(B)** The action potential propagation across the axon leads to ionic transients. The activation of the Na^+^/ATPase and Ca^2+^/ATPase leads to extracellular acidification and extracellular alkalinization, respectively. **(C)** Neurotransmitters are released into the synaptic cleft during propagation of neural response across neurons. **(D)** The two classes of neurochemicals i.e., neurotransmitters and ions can be detected using electrochemical methods such as voltammetry and potentiometry, respectively.

Both ions, such as Na^+^, K^+^, Ca^2+^, Cl^−^ and, neurotransmitters can be used as biomarkers for various neurological disorders (Figure 1). This section describes activity dependent ion and neurotransmitter dynamics under various neurological disorders, which can be used as potential disease biomarkers for use in intelligent neuromodulation systems. The biomarkers listed in Table 1 were selected based on the following criteria, (a) a direct correlation between the biomarker and the clinical symptoms of the neurological disease has been observed, (b) the biomarker also reflects the effect of stimulation and hence can indicate the state of neurological disease after neural stimulation.

**Table 1:** Summary of neurological diseases/conditions and their corresponding potential biomarkers.

**Neurological condition**	**Neural biomarker**	**Recording site**	**References**
Parkinson's Disease	Dopamine	SNc	Lotharius and Brundin, 2002
	Glutamate	SNc	Johnson et al., 2009,
	K^+^, Na^+^, Ca^2+^, Cl^−^	StN	Bittar et al., 2005
Schizophrenia	Dopamine	Prefrontal Cortex Mesolimbic Pathway	Winterer and Weinberger, 2004;Brisch et al., 2014
Cocaine Addiction	Dopamine	Nucleus Accumbens	Groppetti et al., 1973; Volkow et al., 2006
Amphetamine Addiction	Dopamine	Nucleus Accumbens	Groppetti et al., 1973
Stress	Dopamine	Ventral Hippocampus	Pani et al., 2000; Lodge and Grace, 2011
Essential Tremor	K^+^, Na^+^,Ca^2+^, Cl^−^	Ventral Intermediate Nucleus	Krack et al., 2003; Bittar et al., 2005
Chronic Pain	K^+^, Na^+^,Ca^2+^, Cl^−^	Ventral Posterolateral Nucleus	Marchand et al., 2003
		Ventral Posteromedial Nucleus	
Dystonia	K^+^, Na^+^,Ca^2+^, Cl^−^	Globus Pallidus Internus	Krack et al., 2003; Bittar et al., 2005
Dementia	Serotonin	Prefrontal Cortex (Orbitofrontal,	Huey et al., 2006
		Frontal Medial and Cingulate	
		cortices	
Anxiety	Serotonin	^*^	Murphy et al., 2008
Migraine	Serotonin	†	Kowalska et al., 2016
Epilepsy	Serotonin	Raphe Nucleus	Theodore, 2003
		Ipsilateral Thalamus	
		(to epileptic foci)	
Multiple Sclerosis	Serotonin	Lumbar Cerebral Spinal Fluid	Hesse et al., 2014; Malinova et al., 2018
Amyotrophic Lateral Sclerosis	Serotonin	Thoracic Cerebral Spinal Fluid	Sandyk, 2006
Depression	Serotonin	‡	Manji et al., 2001
Alzheimer's Disease	Acetlycholine	Basal Forebrain	Mufson et al., 2008

(*) Current link between serotonin and anxiety is based on measurements of the transporter SERT or the effect of Serotonin Reuptake Inhibitors (SRIs) to ameliorate symptoms of anxiety. (**†**) Link with serotonin was through effect of serotonin receptor (specifically HT_1B_ and 5-HT_1D_) inhibition on migraines and also links with genetic polymorphisms related to serotonin that correlate with migraine propensity. (**‡**) The link between serotonin and depression is through the therapeutic effects of anti-depressants that increase intrasynaptic serotonin and the fact that protocols that deplete monoamines (such as serotonin) have a tendency to precipitate depression. Hence more research is required in order to determine an isolated part of the brain, as yet to measure serotonin in order to provide a direct link.

### 2.1. Ions

The brain is surrounded by extracellular fluid known as the cerebrospinal fluid (CSF), which nourishes the neural tissues with nutrients and performs waste removal. It is mainly composed of water, protein, glucose and ions such as Na^+^, K^+^ etc. A recent review has highlighted covered changes in ion dynamics during onset and duration of seizures (Raimondo et al., 2015). In addition to Na^+^/K^+^, changes in pH have also been detected in glial cells, astrocytes, the cerebellum and the retina in relation to neural activity and also due to electrical stimulation (Makani and Chesler, 2010).

Neurochemical studies in peripheral nerves are very rare, or are at a preliminary stage. They are primarily directed toward detection of ions only. This is because the PNS is composed primarily of axons with cell bodies elsewhere. The earliest known *in vitro* studies demonstrate the presence of extracellular pH change in unmylineated nerve fibers only (Bostock and Grafe, 1985; Bostock et al., 1998). The reported pH changes were in response to electrical stimulation. However, no such work was carried out *in vivo* and in response to physiological stimulation, such as the release of a specific hormone. Recently, we demonstrated *in vivo*, the presence of extracellular pH changes in response to intravenous injection of gut hormone cholecystokinin-8 (CCK) (Cork et al., 2018).

### 2.2. Neurotransmitters

Another viable class of neurochemical biomarkers are neurotransmitters. The following section will examine the neurotransmitters dopamine, serotonin, acetylcholine, and glutamate, each in brief, with respect to their links with neurological disorders.

#### 2.2.1. Dopamine

Within the central nervous system (CNS), the dopaminergic system plays a key role in multiple functionalities including, working memory (Bubser and Schmidt, 1990; Sawaguchi et al., 1990; Sawaguchi and Goldman-Rakic, 1994; Zahrt et al., 1997, reward (Koob, 1992), and locomotion (Whishaw and Dunnett, 1985). The malfunction of this system is linked to a number of neurological disorders including Parkinson's Disease (Lotharius and Brundin, 2002), schizophrenia (Winterer and Weinberger, 2004; Brisch et al., 2014), and addiction (Koob, 1992; Volkow et al., 2006).

In Parkinsonian patients, the substantia pars compacta (SPc) experiences a substantial loss of dopaminergic neurons, which in turn, affects dopamine levels throughout brain regions that receive projections from this area (Lotharius and Brundin, 2002). Dopamine is also used as a reward signal in the brain (Ikemoto, 2007). This system is amplified in amphetamine and cocaine addiction. These substances block dopamine re-uptake and increase dopamine turnover. Furthermore, amphetamine has been shown to directly increase the release of dopamine (Groppetti et al., 1973). There has also been evidence for the role of the dopaminergic system in the stress response. During stress, there is a strong increase in dopaminergic activity (Pani et al., 2000; Lodge and Grace, 2011). Interestingly, a combination of evidence from the above neurological disorders shows a link between dopamine and gastric ulcers (Rasheed and Alghasham, 2012), possibly indicating a link between neurological conditions and the gut, through the gut brain axis. In incidences whereby dopaminergic activity is increased, such as schizophrenia, the incidence of gastric ulcers is significantly lower (Ozdemir et al., 2007).

#### 2.2.2. Serotonin

Serotonin has a modulatory effect across numerous biophysical functions such as arousal (Trulson and Jacobs, 1979), stress (Carhart-Harris and Nutt, 2017), aggressiveness (Lucki, 1998). The malfunction of the serotonergic system has been linked to neurological disorders such as frontotemporal dementia (Huey et al., 2006), epilepsy (Theodore, 2003), multiple sclerosis (Davidson et al., 1977), amyotrophic lateral sclerosis (Sandyk, 2006), depression Manji et al., 2001), and migraines (Kowalska et al., 2016). These disorders are typically characterized by a decreased serotonin level. Interestingly, in cases of depression, the therapeutic effects of increasing the level of serotonin, through administration of serotonin re-uptake inhibitors (SRIs), are often only seen after chronic administration for weeks. This would indicate, that it is in fact, the downstream effects of increased serotonin that produce the therapeutic effect (Manji et al., 2001). There have also been links found between decreased serotonin levels and the pathogenesis of fronto-temporal dementia (Huey et al., 2006).

#### 2.2.3. Glutamate

Glutamate is a key neurotransmitter in the basal ganglia motor circuit and as such, it has been linked with neurological disorders associated with the malfunction of elements of the basal ganglia, such as Parkinson's Disease. Indeed, the administration of glutamatergic receptor antagonists have shown promising results in the treatment of Parkinson's Disease in animal models (Breysse et al., 2002, 2003; Ossowska et al., 2005). The therapeutic effect is thought to be due to two mechanisms; (i) the improvement of adverse motor symptoms of Parkinson's Disease through the direct effect on glutamatergic receptors in the basal ganglia, and (ii) the inhibition of glutamatergic transmission is thought to have a protective effect against neurodegeneration, which may slow down the loss of dopaminergic neurons in the substantia pars compacta (Johnson et al., 2009).

#### 2.2.4. Acetylcholine

The cholinergic system has a key role in the modulation of inflammation in the body. As such, neurological disorders that exhibit inflammation such as multiple sclerosis (Mahad et al., 2015) and Alzheimer's Disease (AD) (Eikelenboom et al., 2000), are thought to be linked to abnormalities in the function of the cholinergic system. In post-mortem analysis of patients with Alzheimer's Disease, there is a clear loss of cholinergic neurons (Mufson et al., 2007), and a significant reduction in cholinergic enzymes, choline acetyltransferase (ChAT) and acetyl-cholinesterase (AChE) (DeKosky et al., 1992). Moreover, much of the cognitive decline that is seen in patients with AD has been attributed to the loss of cholinergic function across the CNS (Mufson et al., 2008).

## 3. Neurochemical Sensors

Neurochemical sensing methods employ primarily bio-electrochemical sensors which are easy to miniaturize and provide label free detection. The underlying chemical reaction involves a redox reaction at the electrode-electrolyte interface (EEI) or through impedance spectroscopy. If a redox reaction is involved, the redox current absorbed by the electrode provides a transduction pathway for the detection and measurement of various analytes. In sensors involving impedance spectroscopy, it involves adhesion or encapsulation of the target biomarker to the surface is needed, resulting in a change in impedance at the EEI.

Traditional chemical detection methods such as microdialysis have more specificity but are less feasible to implant and offer less temporal and spatial resolution (Rodeberg et al., 2017). The Carbon Fiber Microelectrodes (CFM), invented by Gonon et al. (1980), have been widely used in *in vivo* electrochemical recording and are also twenty times smaller than microdialysis probes. This results in less damage to the nervous system when CFM electrodes are inserted (Peters et al., 2004). Due to smaller electrode size, electrochemical techniques are able to offer higher temporal and spatial resolution. Additionally, smaller electrode size also leads to reduced signal distortion due to diffusion when dynamic events are being recorded (Wightman et al., 1988). For instance, it has been demonstrated that it is possible to detect non-evoked dopamine activity associated with electrical neural activity of dopaminergic fibrs (Robinson and Wightman, 2004). This was previously impossible to detect using microdialysis due to diffusion related loss of temporal resolution across the dialysis membrane (Michael and Borland, 2006). Recently, for smaller dimension neural tissue such as nerves, small dimension Iridium-Iridium Oxide (Ir/IrOx) electrodes have been used to perform potentiometric measurements for pH (Cork et al., 2018).

Hence, electrochemical detection of neurochemicals has the advantages of being a microscale, implantable electrode with high spatial and temporal resolution. The trade-off is low biomarker specificity compared to microdialysis techniques. Also, continuous *in vivo* recording is generally accompanied by drift and background activity which needs to be separated in order to extract the signal of interest.

It is important to note that electrochemical techniques are only able to measure change with respect to an unknown baseline. This is a common drawback in all electrochemical methods, as in a static environment, it is difficult to differentiate the contribution due to charging and faradaic currents. Next generation techniques are aiming to measure the basal dopamine level as highlighted in a recent review (Bucher and Wightman, 2015).

**Figure 2:**
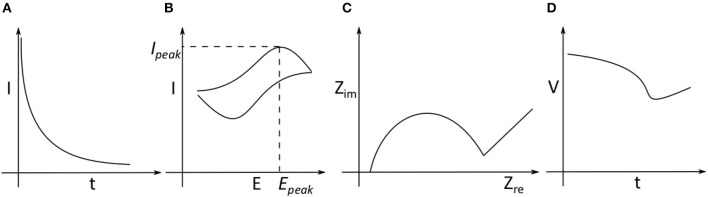
Different electrochemical methods **(A)** Amperometry: where a constant potential difference is applied between the working electrode (WE) and reference electrode (RE). The current between the WE and counter electrode (CE) is monitored as is an indication of the analyte concentration as the reaction progresses. **(B)** Cyclic Voltammetry: The potential difference between the WE, RE is changed periodically and the current between WE and CE is monitored. **(C)** Impedance Spectroscopy: Based on the modality, the impedance of an analyte is measured based on voltage applied between WE, RE and the current through CE. **(D)** Potentiometry: The potential difference between WE and RE is measured without applying any external potential difference.

In this section, we review different electrochemical methods employed in measuring neurochemicals, shown in Figure 2, i.e., amperometry, cyclic voltammetry (CV), electrical impedance spectroscopy (EIS) and potentiometry. Amperometry and CV require three electrodes, consisting of a working electrode (WE), reference electrode (RE) and counter electrode (CE). Potentiometry requires two electrodes consisting only of WE and RE. EIS can be performed using two or more electrodes. The analog front-end circuits required to acquire electrochemical signals have been described in a recent review (Li et al., 2017).

### 3.1. Voltammetry

Voltammetric detection involves the measurement of redox current when a varying cyclic or periodic potential is applied between the WE and RE. The applied potential should be enough to trigger the redox reaction. The current between the RE and CE is proportional to the number of electrolysed molecules, which in turn is indicative of concentration.

#### 3.1.1. Fast Scan Cyclic Voltammetry

In Fast Scan Cyclic Voltammetry (FSCV), a variable voltage, typically having a triangular waveform profile, is applied between the working and reference electrode. The voltage range and scan rate is dependant on the analyte of interest. For example, the parameters used for dopamine are −0.4V to +1.3V at a scan rate of 400 V/s, with a frequency of 10 Hz. The FSCV parameters used for detection of various neurochemicals are listed in Table 2.

**Table 2:** FSCV parameters for detecting various neurochemicals, performed usually at a frequency of 10 Hz.

**Analyte**	**Resting potential (V)**	**Scan rate (V/s)**	**Voltage range (V)**	**References**
Dopamine	−0.4	−0.4 — +1.0/+1.3	400	Venton et al., 2003; Park et al., 2011
Norepinephrine	−0.4	−0.4 — +1.3	400	Park et al., 2011
Serotonin	0	+1.2 —0.6	300	John and Jones, 2006
Oxygen	0	+0.8 —1.4	300	Venton et al., 2003

The fast voltage scan leads to charging of the double layer capacitive-resistive interface at the electrode surface. This leads to large background current which needs to be subtracted in order to resolve the current generated due to the dopamine redox reaction. The cyclic voltammogram profile is unique to each neurotransmitter and the neurotransmitter concentration can be resolved based on current peaks and calibration factors obtained during the standardization process. Calibration could be done using a flow injection system for various analytes such as dompamine pre and post experiment (Venton et al., 2003), to calculate electrode sensitivity. In addition to peak current, other features/parameters that can be used to distinguish voltammograms from different analytes, are rise time and half decay time.

CFM can be pre-treated so that oxidation currents can be resolved at different points under the potential axis. The selectivity of these electrodes can be further improved with the help of a polymer coating called Nafion, which is a sulfonated derivative of teflon (Gerhardt et al., 1984). Fixed anionic sites present in the Nafion membrane help in preventing anionic substances such as urate, ascorbate and acidic metabolites of monoamine neurotransmitters, from reaching the electrode surface and producing interference. This feature also reduces biofouling of the electrode (Turner et al., 1991).

#### 3.1.2. High Speed Chronoamperometry

In chronoamperometry, the WE is held at a constant potential where no reaction is happening and the potential is stepped up to a different potential. This results in the initiation of an electrochemical reaction, upon which the current due to the reaction is measured. There are a variety of pulsed voltage techniques to detect neurotransmitter activity, some of which have been used to study kinetics and clearance mechanisms of serotonin (Daws et al., 2005), and dopamine (Gerhardt and Hoffman, 2001).

#### 3.1.3. Amperometry

In amperometry, the potential of the working electrode is held constant and the current due to the reaction is measured temporally. Amperometry is best suited for conditions where there is a high level of confidence regarding the identity of the analyte being detected (Michael and Borland, 2006). For this reason, it is also used with enzyme-modified electrodes to detect specific non-electroactive species such as glutamate (Kiyatkin et al., 2013), acetylcholine (Sarter et al., 2009) and choline. For nonelectroactive neurotransmitter detection, oxidase enzymes are immobilized on the electrode surface, which, in the presence of target neurochemical, eventually lead to production of an electroactive species. For example, detection of glutamate is performed with glutamate oxidase, where, glutamate is converted to α-ketoglutarate and hydrogen peroxide (H_2_O_2_) (Kiyatkin et al., 2013; Bucher and Wightman, 2015).

### 3.2. Potentiometry

Potentiometry is the measurement of the potential of a solution with the help of two different electrodes, the working electrode which detects the change in chemical reaction and a reference electrode whose potential is known in reference to a standard electrode, such as the Standard Hydrogen Electrode (SHE). In general, the measurement of pH or metal ions can be done using potentiometric methods. The standard, portable pH measurement electrode is the glass electrode.

Previous work in neurochemistry, involving the measurement of pH or ions, was performed using glass electrodes *in vitro* (Endres et al., 1986; Chesler and Kaila, 1992; Makani and Chesler, 2010). However, for *in vivo* measurements, especially in measuring ionic concentrations in PNS, characteristics such as *invasiveness, robustness, small form factor, high sensitivity* and *resolution* are needed. Metal-Metal oxide surfaces such as Iridium oxide (IrOx) can be used to measure pH (Ng and O'Hare, 2015). It can also be fashioned into microelectrodes or microwire electrode and can be used to measure extracellular ionic concentrations *in vivo* in the peripheral nervous system (PNS), such as the vagus nerve (Cork et al., 2018).

### 3.3. Impedance Spectroscopy

Recent work has also shown the potential of using Impedance spectroscopy as a means to detect ionic concentration in the CNS (Machado et al., 2016; De La Franier et al., 2017). A gold substrate is coated with anti-biofouling material to prevent the accumulation of blood or cells. On top of the anti-biofouling layer, 18-6-crown-ether and monoethyleneglycolthiol (MEG-SH) in a 1:10 ratio, respectively, is placed to capture potassium ions (Machado et al., 2016). Another methodology consists of using an oxide layer. This oxide layer is coated with a layer of anti-biofouling material, 3-(3-(trichlorosilyl)propoxy)propanoyl chloride (MEG- Cl). A common issue present in both works is interference from ionic species with similar size such as sodium (Na^+^).

## 4. Closed-Loop: Signal Preprocessing, Decision Making, and Stimulation Dose Selection

The goal of closed-loop neuromodulation is to determine *when?* and *how much?* to stimulate, on the basis of information received directly or indirectly from the neuromodulatory target. Closed-loop neuromodulation can be performed for *prosthetic* or *therapeutic* neurological conditions. This review primarily focusses on *therapeutic* applications, with a focus on using neurochemicals as target biomarkers.

In this Section, different steps involved in implementing a neurochemical based closed-loop neuromodulation system are presented. The first step described is signal pre-processing which is useful in removing baseline drift and identifying symptomatic neurochemical change. This is followed by steps to determine the relationship between symptoms and neurochemical change performed by training set construction and cross validation. It will help the system determine *when* to initiate stimulation. The final step is determining the relationship between electrical stimulation and neurochemical change i.e., stimulation model selection. This will help the system to determine *how much* to stimulate.

Based on functionality and type of control feedback, neuromodulation systems can broadly be divided into five types : *continuous, scheduled intermittent, responsive, adaptive* and *complete closed-loop*, as classified in a recent review by Hoang et al. (2017). *Continuous* neuromodulation is an *open-loop* neuromodulation system, where the stimulation dose is delivered continuously. Adjustments to stimulation dosage is performed by clinicians or care-providers. The feedback in this case are external physiological symptoms and stimulation decisions are made by clinicians. *Scheduled Intermittent* neuromodulation is also a type of *open-loop* where the stimulation is intermittent and no feedback symptom is monitored over time. The stimulations dosage frequency and other parameters such as amplitude, pulsewidth (PW), waveform and frequency are pre-set based on empirical evidence from clinical trials. *Responsive* neuromodulation is a form of partial closed-loop neuromodulation system where the stimulation is initiated automatically based on a physiological biomarker threshold. The stimulation dosage are still pre-set and not tuned in real time. *Adaptive* stimulation is also a form of closed-loop neuromodulation where a single biomarker is monitored. Thresholds and scales on the biomarker are used to determine *when* and *how much* to stimulate. *Complete closed-loop* system consists of monitoring multiple biomarkers answer *when* and *how much* to stimulate.

Both decision making and stimulation dose selection algorithms have to undergo a training phase to enable autonomous operation. The training phase can be conducted on *in vivo* data (Behrend et al., 2009; Trevathan et al., 2015; Bozorgzadeh et al., 2016; Mirza et al., 2019), by recording the neurochemical response to different stimulation parameters. This is followed by cross validation to judge the precision of decision making and stimulation model control algorithms. Prior to *in vivo* training, bench testing using a flow injection system and target analytes may be performed to test electronics and processing system used for data readout and processing (Bozorgzadeh et al., 2016).

**Figure 3:**
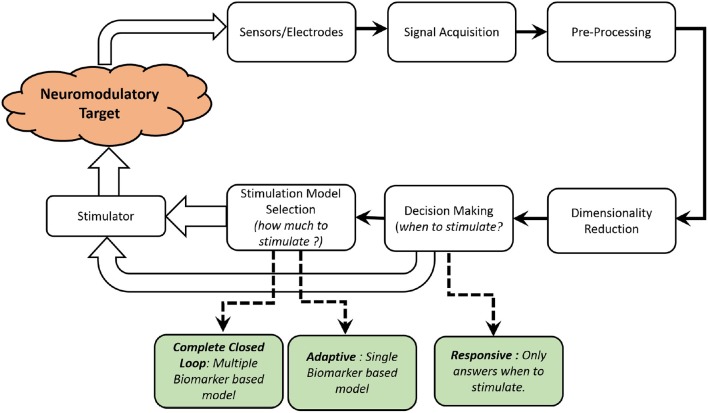
A functional block diagram of a typical closed-loop neurochemical neuromodulation system is shown.

An overview of the different elements in a neurochemical, closed-loop neuromodulation system is shown in Figure 3. As shown in Table 3, there are very limited number of implantable, neurochemical closed-loop systems, as the field is still at a nascent stage. Furthermore, most of the previous work are either *responsive* or *adaptive* closed-loop system with only one biomarker under consideration.

**Table 3:** Review of existing technical platforms for neurochemical closed-loop neuromodulation.

**References**	**Year**	**Neuromodulation** **target application** **(CNS/PNS)**	**Biomarker** **(*when?*)**	**Biomarker** **(*when?*)**	**Sensor type** **(*when?*)**	**Algorithm**	**Outcome**
Cork et al., 2018	2018	VNS	pH	–	Potentiometry	Linear Regression	Non-implantable *in vivo* research device
		(PNS)			using IrOx		*Responsive* operation in
							animal models only
Lee et al., 2017	2017	DBS	Neurotransmitter	Neurotransmitter	FSCV using CFM	ANN^†^	Non-implantable *in vivo* research device
		(CNS)	(Dopamine, Serotonin,	(Dopamine, Serotonin,			*Adaptive* operation in
			Adenosine)	Adenosine)			animal models only
Bozorgzadeh et al., 2016	2016	DBS	Neurotransmitter	–	FSCV using CFM	PCR^*^	Implantable research device
		(CNS)	(Dopamine)				*Responsive* operation in
							animal models only
Grahn et al., 2014	2014	DBS	Neurotransmitter	Neurotransmitter	FSCV using CFM	ANN^†^	Non-implantable *in vivo* research device
		(CNS)	(Dopamine)	(Dopamine)			*Adaptive* operation in
							animal models only
Behrend et al., 2009	2009	DBS	Neurotransmitter	Neurotransmitter	FSCV using CFM	ANN^†^	Non-implantable *in vivo* research device
		(CNS)	(Glutamate)	(Glutamate)			*Adaptive* operation in
							animal models only

† *Artificial Neural Networks*.

* *Principal Component Regression*.

### 4.1. Signal Pre-processing

The chemical signal acquired needs to be pre-processed. Different pre-processing techniques include low pass filtering, downsampling and the removal of drift due to faradaic or background activity. The specifications of different pre-processing elements vary according to the sensing modality and signal characteristics.

#### 4.1.1. Filtering and Downsampling

**Figure 4:**
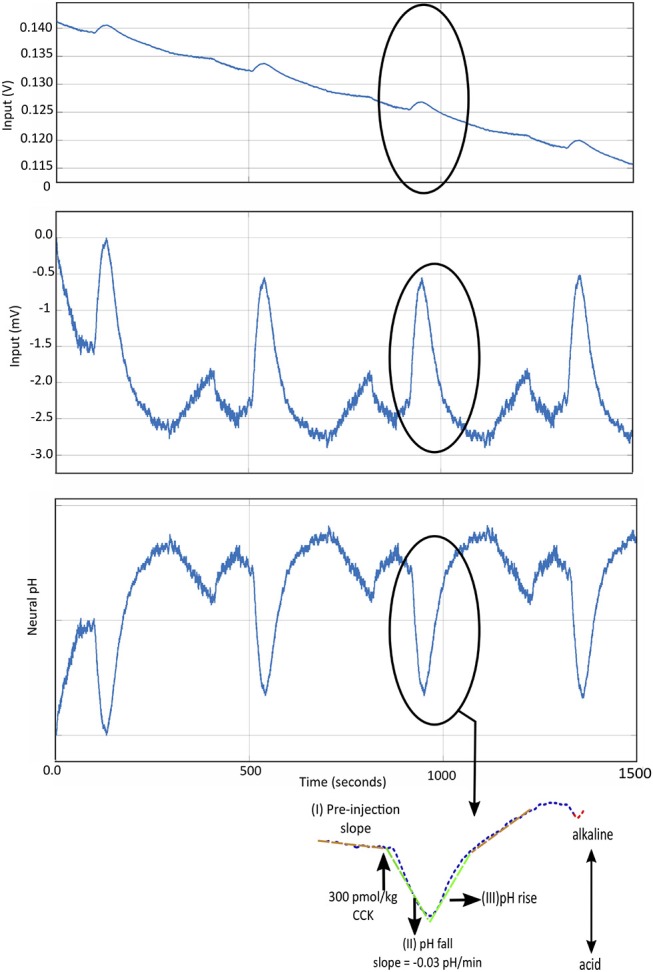
**(Top)** The potentiometric *pH* data recorded using IrOx electrodes, *in vivo*, in the subdiaphragmatic vagus nerve of male Wistar rats. The changes due to CCK are highlighted. **(Middle)** The recorded potentiometric waveform is pre-processed to remove drift using the technique described in Ahmed et al. (2018). **(Bottom)** The ΔpH is determined using the sensitivity of the IrOx pH electrodes, followed by simple linear regression to determine CCK-induced change in neural pH (Cork et al., 2018). This is a demonstration of *responsive* type of intelligent neuromodulation.

Chemical signals are low pass filtered to remove high frequency noise. In FSCV recordings for dopamine, the low pass cut-off is typically set at approximately 100 Hz (Grahn et al., 2014), 1 kHz (Lee et al., 2017), 4 kHz (Bozorgzadeh et al., 2014). In our experiments, where we record pH changes in the sub-diaphragmatic vagus nerve, the pH change induced by CCK (potentiometric measurements) takes about 1–2 min to return to baseline (Figure 4). Hence, in this case, a low pass filter with a -3dB cut-off frequency of 0.1 Hz is enough to remove any high frequency interference including any line interference (50Hz/60Hz). In order to maintain high resolution, some solutions implement sigma-delta (ΣΔ) analog to digital converters (ADC) (Bozorgzadeh et al., 2016; Lee et al., 2017). This results in oversampling of data which is further sampled down using Cascaded Integrator-Comb (CIC) filter as in Bozorgzadeh et al. (2016), to reduce data throughput.

#### 4.1.2. Background Subtraction and Drift Removal

The current recorded whilst recording FSCV to sense neurotransmitters, is a combination of faradaic and background current. Similarly, during potentiometric recording, there is interference from background potentiometric changes and also a consistent, sometimes unidirectional drift, due to changes in Open Circuit Potential (OCP) of the potentiometric electrode. Hence, it is essential to remove large changes in background signal, before performing dimensionality reduction or pattern recognition, to identify signatures related to the neural activity being monitored.

A common technique in FSCV drift reduction is the subtraction of recorded current with a short recorded window of the previous current. This is possible because it has been observed during *in vivo* recordings, that FSCV current signatures due to dopamine transients occur in the range of 2–3 ms. Secondly, it has been observed that background current is typically stable over few seconds (Bozorgzadeh et al., 2016). Hence, the background current can be recorded, averaged over few scans (two or four scans) and then subtracted to remove any background activity as described in Bozorgzadeh et al. (2014) and Bozorgzadeh et al. (2016). Other solutions such as the WINCS Harmoni platform also implement background subtraction (Lee et al., 2017). For pre-processing of CCK induced pH changes, a resource efficient architecture was recently described, where the recorded data is down-sampled and slow, non-linear drift was removed in real-time (Ahmed et al., 2018).

### 4.2. Dimensionality Reduction

Data collected during neurochemical recordings are highly complex with multiple variables affecting readings, hence a multivariate dimensionality reduction technique needs to be utilized in order to ensure accurate analysis of the data and for detection of target neurochemical signature. Principal Component Analysis (PCA) has been widely used as a preferred technique for dimensionality reduction in a number of neurochemical FSCV applications (Keithley et al., 2010). PCA combined with inverse-least squares regression, known as Principal Component Regression (PCR), is used to make predictions regarding the concentration of target neurochemical analyte (Heien et al., 2004, 2005; Keithley et al., 2005, 2010; Keithley and Wightman, 2011; Bucher et al., 2013; Bozorgzadeh et al., 2016). PCA is a mathematical technique which, from a dataset of possibly correlated variables, identifies a set of vectors which are linearly uncorrelated (mutually orthogonal), called “principal components” (PCs). The application of PCA to *chemometrics* involves a four step procedure: (a) signal identification (b) training set construction, (c) generation and selection of relevant PCs, (d) cross validation of PCs.

#### 4.2.1. Signal Identification

Signal identification consists of determining the signal characteristics which are directly correlated to changes in the target neurochemical analyte. The steps involved in signal identification may consist of *in vivo* experiments, followed by signal processing and statistical steps such as ANOVA to ensure reproducibility (Keithley et al., 2005; Cork et al., 2018). After identification of the neurochemical response, it is crucial to model the relationship between (a) the neurochemical response and physiological symptom under study, (b) the relationship between the electrical stimulation and the neurochemical response to it. This is achieved through a combination of mathematical modeling and machine learning techniques described later in this section.

#### 4.2.2. Training Set Construction

The training matrix is generated by combining the temporal signatures of changes in target analyte that were observed electrochemically. For multivariate classification, background changes due to electrode drift or changes in interferring neurochemicals are also considered (Bozorgzadeh et al., 2016).

**Figure 5:**
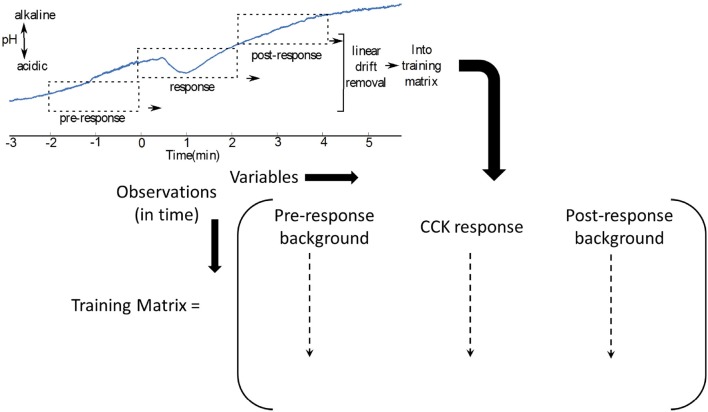
The training matrix can be constructed for as shown, for CCK induced pH changes in the vagus nerve (Mirza et al., 2017).

In Figure 5, where the interferents are not known, the pre-injection and post injection waveforms could be used as two types of background signal, in addition to the response to generate a training matrix (Mirza et al., 2017).

#### 4.2.3. Generation and Selection of Relevant PCs

The PCs are generated using Single Value Decomposition (SVD) (Equation 1). The commands listed in MATLAB are listed below.

(1) $$\begin{array}{l} \left[U,S,V\right]=svd\left(A\right) \end{array}$$

A *scree* plot can be used to determine the variance captured by each PC. The PCs which capture maximum variance are relevant and hence, are retained.

(2) $$\begin{array}{l} Uc=U\left(:,1:k\right) \end{array}$$

where k is the number of relevant PCs. The decision to retain relevant PCs can be used to generate projections (*A*_*proj*_) of the training matrix (A) onto the PCs:

(3) $$\begin{array}{l} A_{proj}=A\times Uc \end{array}$$

where U is the PC matrix, composed of PCs generated from the training matrix. If PCR is used, then a regression matrix based on the dose response nature of the analyte is also generated as shown in Equation (11). After the PC matrix is calculated, the next step is cross validation.

#### 4.2.4. Cross Validation of PCs

Cross validation involves determining the robustness and applicability of the training set. This is required to ensure the PCs are sufficient to perform dimensionality reduction in real time. There are a number of methodologies which can be utilized to cross-validate PCs (Keithley et al., 2005, 2010), one such method being residual analysis, which is described below. The residual of a data set is defined as the difference between actual data and data projected using the PCs. A parameter *Q*_*t*_ is defined as the difference in the square value of the actual and projected sample values, *a*_*i*_ and ã_*i*_, respectively.

(4) $$\begin{array}{l} Q_{t}=\overset{N}{\underset{i=1}{\sum }}\left(a_{i}^{2}-{ã}_{i}^{2}\right)=\left(a_{1}^{2}-{ã}_{1}^{2}\right)+\ldots +\left(a_{N}^{2}-{ã}_{N}^{2}\right) \end{array}$$

where N is the total number of datapoints. *a*_*i*_ and ${ã}_{i}^{2}$ are actual and projected data values, respectively, for the *i*^*th*^ sample. A significance threshold, Q_α_, is set so that the residual for each dataset, Q_*t*_, does not exceed Q_α_. For the training set to be considered robust, Q_*t*_ must be less than Q_α_ for all data in the validation set. When Q_*t*_ is greater than Q_α_, for a specific dataset, it indicates that the variance in the input data is not appropriately captured in the training set. It can indicate the data is non-deterministic and can lead to false positives. Q_α_ is calculated using the following formula (Keithley et al., 2010) :

(5) $$Q_{\alpha }={⊖}_{1}\left[\frac{c_{\alpha }\sqrt{2{⊖}_{2}h_{0}^{2}}}{{⊖}_{1}}+1+\frac{{⊖}_{2}h_{0}(h_{0}-1)}{{⊖}_{1}^{2}}\right]$$

where *k + 1* to *n* is the number of discarded PCs, k is number of retained PCs and n is the total number of PCs generated.

(6) $$\begin{array}{l} \gamma =S^{2} \end{array}$$

where γ is the sum of the square of the projected datapoints from the training matrix and S is the singular value matrix generated from Equation (1).

(7) $$\begin{array}{l} {⊝}_{i}=\overset{n}{\underset{j=k+1}{\sum }}\gamma_{j}^{i} \end{array}$$

(8) $$\begin{array}{l} h_{0}=1-\frac{2{⊝}_{1}{⊝}_{3}}{{⊝}_{2}^{2}} \end{array}$$

where, Q_α_ denotes an upper limit on the random error to be tolerated. In Equation (5), for c_α_ = 1.645 or 2.326 Q_*t*_ will be greater than Q_α_ if 95% or 99%, respectively, of the dataset are due to random noise. More details are provided in Keithley et al. (2010).

### 4.3. Decision Making

This step involves determining *when* to stimulate. The decision when to stimulate could be based on a number of criteria. It could be (a) *threshold based*: stimulation is initiated when a specific neurochemical signature is detected and the response has reached a specific threshold (Bozorgzadeh et al., 2016) or (b) *response based*: where only the presence of a response is enough to trigger stimulation (Cork et al., 2018). In order to implement this, various statistical techniques could be utilized. Techniques such as Simple Linear Regression can be used in *response based*, univariate, decision making. Inverse least square regression in combination with PCA can be used to perform multivariate analyte concentration and a decision regarding *when* to stimulate can be made, based on the threshold of analyte concentration. In this section, we describe a multi-variate decision making model (Bozorgzadeh et al., 2016).

#### 4.3.1. Simple Linear Regression

As shown in Figure 4, certain characteristics of the recorded neurochemical signal can be extracted and a simple linear regression model can be fitted on it to extract and identify a signature. This is a type of *univariate* detection method where only one variable is considered to affect the neurochemical signal. This technique was successfully demonstrated to implement a responsive closed-loop neuromodulation technique in an *in vivo* experimental setup. However, since this technique is a univariate approach, it is sometimes susceptible to false positives, hence it is best to limit this to stable *in vivo* experimental environment and not to extend it in an implant.

#### 4.3.2. Inverse Least-Squares Regression

In this process, the regression matrix generated is used to estimate the concentration of the analyte. Different concentrations of analyte can result in different amplitude of neurochemical response peaks. Based on a preset threshold for analyte concentration, stimulation can be initiated. This process was first described in Keithley et al. (2005), followed by an implementation on a System-On-Chip (SoC) in Bozorgzadeh et al. (2016).

The regression matrix is based on the dose response to the analyte concentration (Bozorgzadeh et al., 2016). The regression matrix, in combination with the projected data set from PCA, can be used to predict the concentration of the analyte. This is described in the equation below :

(9) $$F\;=C\times A_{proj}\times (A_{proj}^{\prime }\times A_{proj})$$

(10) $$\begin{array}{lll} D_{proj} & = & D_{u}\times U_{c} \end{array}$$

(11) $$\begin{array}{lll} \left[C_{A}C_{B1}C_{B2}\right] & = & D_{proj}\times F \end{array}$$

where *C* is the concentration matrix i.e., a diagonal matrix with concentration values of each analyte considered in a multivariate model, *A*_proj_ is the projection matrix defined in Equation (3), D_*u*_ is the real time data, D_*proj*_ is the projection of the real time data on the PCs, *U*_*c*_ is defined in Equation (2). *F* is the projection matrix and *C*_*A*_, *C*_*B*1_, *C*_*B*2_ are the projected concentrations based on inverse least-squares regression.

### 4.4. Model Selection

The primary goal of model selection is to determine optimal electrical stimulation parameters based on the relationship between stimulation parameters and the target neurochemical biomarker. It is a crucial step toward *adaptive* or *complete closed-loop* neuromodulation to determine the stimulation dose. The relationship between stimulation parameters and neurochemical biomarkers is established based on experimental data and mathematical modeling. Various linear (Behrend et al., 2009) and non linear (Grahn et al., 2014; Lee et al., 2017) modeling techniques have been utilized previously to develop stimulation models. The model selection could be different based on whether the neuromodulatory target is located in the CNS or PNS. In this section, we will describe briefly, one linear and two non-linear stimulation models.

#### 4.4.1. CNS: Stimulation Evoked Release and Uptake of Neurotransmitters

DBS of specific areas in the brain is considered an effective therapy for the treatment of Parkinson's disease. For CNS disorders, the neurochemical biomarkers generally under consideration are dopamine, serotonin (Grahn et al., 2014; Lee et al., 2017) and glutamate (Behrend et al., 2009). In closed-loop DBS, the goal is to maintain a specific concentration of neurotransmitters (Lee et al., 2017). Several publications demonstrate possible techniques for choosing appropriate stimulation parameters (Wu et al., 2001; Behrend et al., 2009; Grahn et al., 2014; Walters et al., 2014; Lee et al., 2017). Behrend et al. (2009) use model equations, whereas Artificial Neural Network (ANN) is used in Lee et al. (2017)and Grahn et al. (2014). In this paper, we principally describe stimulation model selection based on linear or non-linear modeling techniques described in Behrend et al. (2009), Lee et al. (2017), and Grahn et al. (2014), respectively.

In Behrend et al. (2009), the concentration of glutamate recorded close to the sub-thalamic nucleus (StN) was modeled as a function of electrical stimulation. The parameters of electrical stimulation used in Behrend et al. (2009), were fixed (stimulation current ≈ 100 μA, a stimulation pulsewidth of 1 ms for 100 Hz stimulation frequency and 0.67 ms for 150 Hz stimulation frequency). A second order model equation, based on Auto Regressive eXogenous (ARX) fitting was developed, shown in Equation (12). This was validated using cross correlation between simulated and recorded concentrations of glutamate, for varying stimulation durations in a rat model.

(12) $$\begin{array}{l} A\left(q\right)\times y\left(t\right)=B\left(q\right)u\left(t\right)+\varepsilon \left(t\right) \end{array}$$

where, y(t) represents the glutamate concentration, u(t) corresponds to the input stimulation parameters and ε(t) corresponds to the stochastic error. For each set of stimulation parameters (i.e., two different sets of stimulation pulsewidth and frequency), the stimulation was switched on randomly to accurately capture the dynamic response of the system (Behrend et al., 2009).

The limitations of the stimulation model included limited visibility into the effects of changing stimulation currents, which is a crucial parameter to consider in DBS. Also, the model will benefit by selecting a wider range of pulsewidths and frequency. Furthermore, in order to further develop the therapy, the effects of stimulation on concentrations of other neurotransmitters such as dopamine and GABA are not modeled. The model itself is univariate, hence it does not consider interference from other neurochemicals with similar oxidation potential. The model also does not take the non-linear nature of neurochemical responses, into consideration. The univariate and linear transfer function, described in this model, needs to be expanded to ensure the model is applicable in a long-term implant.

The stimulation model, described in Behrend et al. (2009), is based on normalized values of glutamate concentration across animals. This was due to large variance in absolute glutamate concentrations across animals and also partly due to the limitation of neurochemical measurement based on electrochemical methods, which are only able to measure the change in analyte concentration only. However, the range of control on a normalized analyte concentration, in this case glutamate, is crucial. In Behrend et al. (2009), results suggest a normalized concentration range between 0.4 and 1.0 were set as control thresholds.

In Lee et al. (2017), the stimulation model was adopted from Trevathan et al. (2015). It is based on modeling dopamine kinetics due to electrical stimulation, using two different frameworks, ANN and time-series approach using Volterra kernels. Volterra kernels are particularly useful to capture the short-term and long-term effect of stimulation parameters (input) on neurochemical responses (output), in non-linear systems. Hence, they are useful in capturing the hysteresis effect i.e., effect of previous electrical stimulation events on present neurochemical responses (Trevathan et al., 2015). On the contrary, ANNs are better suited for compartmental modeling of input/output relationship between stimulation parameters and stimulation-evoked neurochemical release (Walters et al., 2014, 2015). The experimental data was obtained by stimulating the medial forebrain bundle (MFB) in rats and recording neurochemical data (Trevathan et al., 2015). A similar method was also adopted in the striatal and ventrotegmental area / substantia nigra pars compacta (VTA/SNc) of swine and non-human primate (NHP) (Trevathan et al., 2015). The stimulation parameters under consideration were stimulation amplitude (current) and pulsewidth. The stimulation duration was randomly selected to be either 0.5 or 2.0 s, to capture the dynamics of dopamine, while attempting to avoid hysteresis.

In Grahn et al. (2014), a combination of non-linear regression, computational modeling and constrained optimization was used for linking stimulation parameters with stimulation evoked dopamine responses during the experimental phase. The target stimulation electrode was placed in the medial forebrain bundle (MFB), the FSCV recording electrode was placed in the striatum and a reference silver-silver chloride electrode was placed in the contralateral cortex, to record dopamine concentration. The stimulation parameters (current, pulsewidth, frequency) were varied to record evoked dopamine responses. This dataset consisting of the stimulation parameters and their corresponding evoked dopamine responses were modeled as a combination of a 7th degree polynomial and 2nd order exponential mathematical models. The parameters in the model i.e., 8 for polynomial, 4 for exponential, and corresponding stimulation parameters were presented to an ANN. The ANN was a double feedforward ANN with sigmoidal and linear transfer funtions (Lujan and Crago, 2009). Later, in order to demonstrate closed-loop neuromodulation, the stimulation parameters required for sustaining dopamine responses at desired levels were predicted using ANN. Hence, to summarize the build-up of ANN, the inputs consisted of three stimulation parameters (stimulation frequency, pulsewidth and stimulus amplitude/current) and system outputs consisted of 12 model parameters.

The ANN consisted of 150 hidden neurons, the initial weights and biases are based on 10 different initial conditions and 10 corresponding ANNs were trained on 80% of the data (Levenberg-Marquardt algorithm). The remaining 20% of the data were used to simulate ANNs and identify those with the lowest generalization error. Constrained optimization was added to the ANN model to minimize stimulation energy and to eliminate mathematical redundancies. In order to determine the accuracy of the model, the predicted stimulation parameters and the measured dopamine levels evoked due to predictive stimulation was compared with the desired simulation results. The root mean squared error between measured and desired dopamine levels was determined, followed by least-squares regression analysis to determine dependencies between actual and desired dopamine levels were theni used in order to identify sources of error such as drift. Results in Grahn et al. (2014), from four rats, suggest that the computational and predictive models of stimulation-evoked dopamine levels can be adjusted using predicted stimulation parameters (R^2^ = 0.8).

#### 4.4.2. PNS: Neurochemical Recordings and Stimulation Evoked Compound Nerve Action Potentials (CNAPs)

The goal of stimulation model selection in PNS neuromodulatory therapeutic applications is to either inhibit or enhance nerve fiber activity. Peripheral nerve activity can be classified into (a) neural mass activity, (b) CNAPs and (c) Neurochemical ionic activity. Neurochemical recording can be utilized reliably to detect specific physiological events and initiate stimulation (Cork et al., 2018). However, stimulation dose tuning can be achieved through monitoring electrical activity alone. A method to estimate the properties of individual fibers is through interrogative stimulation and recording. A stimulation protocol and method for high resolution CNAP recording on vagus nerve is described in Mirza et al. (2018).

**Figure 6:**
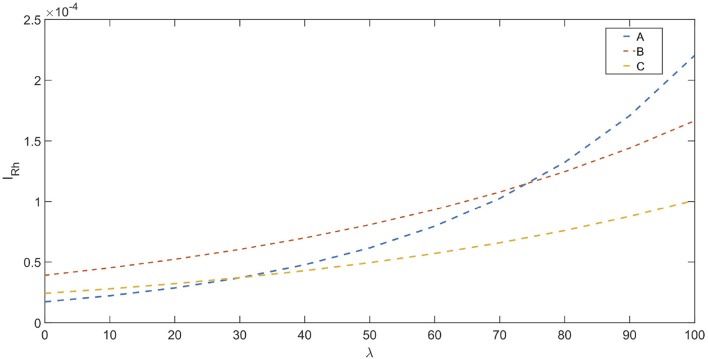
NAP profiles for different fiber types : A, B, and C based on Ward et al. (2015), the rheobase current (I_*Rh*_ in A) is depicted vs. the percentage fiber activation(λ).

In Ward et al. (2015), an effort has been made to determine stimulation dosage based on CNAPs. This involves determining a *nerve activation profile* (NAP) for each fiber type. The nerve activation potential is based on an exponential relation between rheobase current and normalized CNAP amplitude. Stimulation dosage (stimulation current) can be chosen appropriately based on NAPs. The relationships are shown below (Ward et al., 2015) :

(13) $$\begin{array}{l} I_{Rh_{A}}=e^{0.0255\lambda -10.97} \end{array}$$

(14) $$\begin{array}{l} I_{Rh_{B}}=e^{0.0145\lambda -10.15} \end{array}$$

(15) $$\begin{array}{l} I_{Rh_{C}}=e^{0.0143\lambda -10.63} \end{array}$$

where, *I*_*Rh*_ is the rheobase current. λ = $\frac{V_{cnap}}{V_{cnap\text{\_}max}}$ × 100, which represents the percent maximal activation of nerve fibers. The NAP for different fibers are shown in Figure 6.

In existing solutions, such as Behrend et al. (2009), Grahn et al. (2014), and Lee et al. (2017), model selection was performed using ANN. Implementation of ANN on-chip or SoCs can be resource and power intensive. To enable on-chip implementation of *model selection*, it is important to consider simplification of the stimulation control model by balancing the trade-off between complexity of the model and stimulation goals.

Alternatively, a better approach to develop implantable solutions, especially for PNS applications, will be to determine a mathematical model as described in Ward and Irazoqui (2010) and Behrend et al. (2009). Linear or polynomial models can be implemented on-chip and optimized to be resource and power efficient. Another approach will be to design stimulators which can perform selective activation of fibers by modifying the traditional stimulation waveforms (Joseph and Butera, 2011; Patel and Butera, 2015).

## 5. Discussion

Closed-loop neuromodulation is likely to improve system performance and clinical outcomes. However, the challenges involved in implementing closed-loop neuromodulation consists of identification of appropriate biomarkers, identifying the recording and stimulation loci and the choice of neuromodulatory paradigm i.e., excitation/inhibition of neural activity or regulation of neurotransmitter concentration. A neurochemical monitoring modality for closed-loop neuromodulation is promising, as it provides higher resolution in terms of neural events, better Signal-to-Noise ratio and less interference in comparison to electrical recordings of neural signals.

This review highlights that, neurochemical closed-loop neuromodulation systems benefit from higher specificity and less interference for *therapeutic* neuromodulation applications. However, there is a clear lack of implantable, closed-loop neurochemical neuromodulation systems that are available commercially. The lack of neurochemical closed-loop neuromodulation is due to various reasons.

One of the limitations of neurochemical monitoring is ensuring direct contact between the neurochemical sensor and neurochemical molecules. For this reason, the sensors need to be inserted into neural tissue and hence have greater susceptibility to bio-fouling. It is a long standing challenge to produce neurochemical sensors whose sensitivity lasts longer *in vivo*. CFM has been widely used to perform neurochemical voltammetric measurements and has been shown to work reliably up to 4 months after chronic implantation (Clark et al., 2010). Carbon fiber microelectrodes can also be coated with CNTs (Swamy and Venton, 2007), which increases its resistance to the adhesion of common biofouling agents such as 5-HIAA. CNT-based microelectrodes are able to increase selectivity and sensitivity of FSCV measurements at high speeds (Bucher and Wightman, 2015). Especially, polyamide-coated fused silica CFM electrode have been shown to last *in vivo* for approximately 25 days without any loss in sensitivity, albeit a small loss in temporal resolution (Clark et al., 2010). Efforts to tackle this challenge further include developing anti-biofouling coatings on the sensor (Blaszykowski et al., 2015; Machado et al., 2016). However, the lifetime of *in vivo* ion-selective electrodes needs to be investigated further.

A very promising line of research has been pursued by Thompson's group at the University of Toronto, who are developing anti-biofouling coatings for *in vivo* sensor applications (Sheikh et al., 2012, 2015). Furthermore, the same group have developed an anti-biofouling coating for potassium sensors, which will include neuro-chemical recording (Thompson, 2017).

Another important effort in sensor fabrication is directed toward reducing the size of the array while increasing recording locations. This will increase spatial resolution leading to better recording of neurotransmitter concentrations within the extracellular/intracellular environment. In this direction, research is progressing to explore the development of carbon-nanotube (CNTs) or carbon-nanofiber (CNF) based microelectrode array (MEA) which are compatible to be used for neurochemical measurements (Bucher and Wightman, 2015). In order to capture neurotransmitter activity from extracellular or exocytotic neural environment in the brain, the microelectrode array (MEA) pitch needs to be between ~10−20μ*m* to enable interfacing with individual neurons (Kishida et al., 2011). The latest neurochemical MEA consists of 36 microelectrodes within an area of 40 μm × 40 μm (Bucher and Wightman, 2015).

The majority of neurochemical recording in the CNS, reported earlier, is focussed on neurotransmitters. However, the relationship between stimulation parameters and stimulation-induced change in neurochemical concentration, may vary over time. This change could be due to neuro-plasticity or change in electrode-electrolyte interface, leading to re-adjustment of stimulation models on a regular basis, to adjust stimulation dosage accurately.

An important aspect of neurochemical recording is the interference from other neurochemicals. One of the common interferents is pH change, which produces similar profile of voltammograms to dopamine. Hence, it is important to subtract contribution due to pH in order to identify dopamine specific cyclic voltammogram. Another common interferent is ascorbate, which also has similar oxidation potential as dopamine but different voltammogram profile. A multivariate classification model can be used for distinguishing signal contribution due to target neurochemical signals and interferents. Furthermore, electrode design can also be utilized to reduce cross-talk between different neurochemical signals. It was shown that CNF performs better than glass carbon electrode in isolating signal contributions due to different neurotransmitters (Rand et al., 2013). Different neurotransmitters which show similar oxidation potentials using glass carbon electrodes, show different oxidation potentials when CNF is used.

Also, in diseases where there isn't a clear relationship between neurotransmitters and disease symptoms, there may exist a clear relationship between electrical neural signals and symptoms. In such cases, it is better to monitor neural ionic concentrations, such K+, Na+, or H+ which are also directly correlated with electrical neural signals (Makani and Chesler, 2010). This will ensure high specificity biomarker recording. Another fact to note is that the recent work in neurochemical monitoring mostly focusses on healthy animal models (Chang et al., 2012; Lee et al., 2017) and very limited work has been done in humans. Two human studies were performed by Kasasbeh et al. (2013) and Chang et al. (2012), in which no adverse effects to patient health were reported in the short-term. Also, no short-term reduction in DBS treatment efficiency was observed.

In existing neurochemical neuromodulation systems, the training step and in some cases, the entire *decision making* and *model selection* algorithm is implemented off-chip. Although, this works for research applications, it restricts patient mobility in a future implantable solution. In order to achieve local or on-chip machine learning capabilities, it is important to reduce complexity of the neuromodulation algorithm without sacrificing stimulation goals. Commercial solutions, such as those from ARM and Qualcomm, are also focussed on developing resource efficient, artificial intelligence on SoC (Desoli et al., 2017; Moons et al., 2017).

Another important aspect of achieving therapeutic efficiency is stimulating at the optimal location. When DBS is used for treating Parkinson's, the stimulation locus is generally the sub-thalamic nucleus/globus palliadus internus (StN/Gpi) (Hitti et al., 2019). For VNS, various locations have been used for different applications such as stimulation of left or right cervical vagus nerve for epilepsy (Boon et al., 2001), subdiaphragmatic vagus nerve for obesity (Ikramuddin et al., 2014), auricular branch of the vagus nerve (Clancy et al., 2014).

The WINCS platform has demonstrated that it is possible to develop an adaptive neuromodulation system based on a single neurochemical biomarker. However, the long term efficacy of a single biomarker based solution needs to be determined. A solution to better understand the success and, ultimately, improve closed-loop neuromodulation therapy is the addition of electrophysiological recording. Recently, an electrode was developed by Vajari et al. (2018), which incorporates the ability to record both neurochemical, neuro-electrical recording and electrical stimulation. This is a desirable addition for CNS based therapy, but is necessary for complete closed-loop in a PNS based closed-loop neuromodulation system (Mirza et al., 2017, 2019).

## 6. Conclusion

This review is focused on highlighting the benefits and challenges of using a neurochemical biomarker for intelligent neuromodulation. It has also outlined the different elements required to implement neurochemical closed-loop neuromdoulation as an implantable solution. The first step toward developing an intelligent neuromodulation system is identifying an appropriate biomarker, such as a neurochemical biomarker and a corresponding stimulation/recording site. The second step is to develop a reliable sensing methodology, sensor and data acquisition system. The third challenge is to implement the pre-processing and intelligent neuromodulation algorithms on-chip or locally in a single integrated SoC.

For CNS based applications, where neurotransmitters are the target biomarkers, FSCV is often chosen as a reliable detection technique and CFM is the preferred electrode for this application. Emerging techniques include impedimetery and potentiometry for detection of ionic concentrations, in both CNS and PNS. For potentiometric sensing, IrOx sensors can be used reliably for sensing pH. The sensitivity, selectivity and longevity of the sensors described in this paper can be improved through coatings such as, Nafion-CNT.

There are primarily two technical challenges that need to be addressed in order develop a neurochemical closed-loop system for long-term, chronic therapeutic efficacy studies. The first is development of an implantable chemical sensor, with a reliable sensitivity and resolution in the long term. The second challenge is implementing processing algorithms on-chip for stimulation decision making (*when?* to stimulate) and stimulation model selection (*how much?* to stimulate).

## Author Contributions

KM and CG wrote the manuscript. KN and CT supervised and reviewed the manuscript.

### Conflict of Interest Statement

CT holds a patent on chemical monitoring of the vagus nerve (US9055875 B2). The remaining authors declare that the research was conducted in the absence of any commercial or financial relationships that could be construed as a potential conflict of interest.
